# Pre-eruptive intramural resorption in unerupted teeth: a cone-beam computed tomography evaluation of prevalence and related factors

**DOI:** 10.1007/s00784-024-05677-4

**Published:** 2024-04-27

**Authors:** Supak Ngamsom, Tawepong Arayapisit, Phanit Asavanamuang, Raksayam Anurakwongsri, Kittikarn Sonthinane, Kornkamol Kretapirom

**Affiliations:** 1 Department of Oral and Maxillofacial Radiology, Faculty of Dentistry, Mahidol University, 6 Yothi rd, Thung Phaya Thai, Ratchathewi, Bangkok, 10400 Thailand; 2 Department of Anatomy, Faculty of Dentistry, Mahidol University, 6 Yothi rd, Thung Phaya Thai, Ratchathewi, Bangkok, 10400 Thailand; 3 Mahidol International Dental School, Faculty of Dentistry, Mahidol University, 6 Yothi rd, Thung Phaya Thai, Ratchathewi, Bangkok, 10400 Thailand

**Keywords:** Pre-eruptive intramural resorption, Pre-eruptive intracoronal resorption, Cone-Beam computed tomography, Unerupted tooth, Tooth resorption

## Abstract

**Objective:**

Pre-eruptive intramural resorption (PEIR) is defined as an abnormal, well-circumscribed radiolucency within the coronal dentin of the tooth, which is often overlooked in plain radiographs. This study aimed to investigate the prevalence of PEIR and its related factors using cone-beam computed tomography (CBCT).

**Methods:**

CBCT images of 590 unerupted teeth were evaluated for the presence of PEIR, location of PEIR, number of lesions in the affected tooth, PEIR score, tooth angulation, tooth position, and pericoronal space. Binary logistic tests were used to analyze the association between the characteristics of PEIR and the patient’s demographic data and related factors.

**Results:**

The tooth prevalence of PEIR was 13.6% among unerupted teeth. However, it was noteworthy that 19.2% of the unerupted teeth with PEIR were planned to be kept. PEIR was significantly associated with transverse (*p* = 0.020), inverted-angulated (*p* = 0.035), and centrally-positioned teeth (*p* = 0.043). The severity of PEIR was more pronounced in teeth with distal (*p* = 0.019), lingual (*p* = 0.023), or inverted-angulated (*p* = 0.040) positions, and in the absence of pericoronal space (*p* = 0.036).

**Conclusion:**

PEIR should be suspected in transverse, inverted-angulated, centrally positioned unerupted teeth, particularly in molars, with no pericoronal space. Further monitoring through CBCT is recommended in such cases.

**Clinical relevance:**

The management of unerupted teeth does not always involve surgical removal. Instead, they could be utilized for artificial eruption or tooth transplantation. The present study emphasizes the significance of early detection of PEIR. Clinical recommendations for screening PEIR in unerupted teeth are also proposed, which can be applied to routine plain radiographs.

## Introduction

The management of unerupted teeth does not always necessitate surgical removal; alternatively, they can be considered for artificial eruption or tooth transplantation. In cases involving such teeth, resorptive abnormalities including pre-eruptive intramural resorption (PEIR) may be present, but are often overlooked due to limited information and awareness, since PEIR can only be detected radiographically. However, initial lesions are frequently unnoticed until they have progressed extensively, with no established screening protocol in place on plain radiographs.

PEIR is defined as an abnormal, well-circumscribed radiolucency within the coronal dentin of the tooth [1, 2]. It has been associated with periapical inflammation of primary teeth, dental caries, and localized developmental defects of dentin [3]. However, these theories have not consistently received support from other researchers. Despite this, local factors, particularly ectopic positioning, appear to play a role in the etiology of PEIR. Ectopic positioning can lead to increased pressure, damaging the integrity of the reduced enamel epithelium and allowing resorptive cells to function and thereby, resorbing the dentine [4]. Until now, Investigations into other local factors, such as abnormal positioning and the pericoronal space of the unerupted tooth, have been limited. The most widely accepted theory, nowadays, is the intra-coronal resorption of dentin, occurring when crown development is interrupted. This process is led by resorptive cells, including chronic inflammatory cells, osteoclasts, and chronic multinucleated giant cells. Both external and internal resorptions have been considered to be one of the potential causes of PEIR; however, the factors that activate resorption are currently unknown [1].

PEIR can manifest in either a static or progressive nature. In cases of progressive PEIR lesions, close observation or immediate treatment may be necessary [5]. The commonly employed diagnostic tools for diagnosing and monitoring these lesions include periapical, bitewing, and panoramic radiographs. Although occasionally used, cone-beam computed tomography (CBCT) compensates for the limitation of detecting these lesions extensively in the bucco-lingual dimension and assessing pulpal involvement.

Treatment options for PEIR varied, ranging from restorative intervention either before or after eruptions, to extraction of the tooth in extensive cases [4, 6]. A follow-up approach was recommended for lesions that did not exceed half of the dentine thickness. In cases where the lesion demonstrated a non-progressive nature that would not endanger the pulp, a restoration after eruption might be deemed appropriate [7, 8]. For larger lesions that were not close to eruption and exhibited a progressive nature, immediate surgical treatment was advisable [4, 9]. In cases involving pulp exposure, viable options including pulp capping with calcium hydroxide, or mineral trioxide aggregate (MTA), and Biodentine in vital pulp therapy were suggested [10]. However, when the lesion was symptomatic or had progressed extensively, extraction of the affected tooth should be considered immediately after eruption.

CBCT is not only utilized to investigate the extent of PEIR lesions in all directions but also to examine possible predisposing factors such as ectopic positioning and pericoronal spaces. This comprehensive approach allows for further exploration of prevalence and invasion characteristics, including defective scores and the number of lesions within the same tooth. To our knowledge, investigations into these factors are unprecedented; however, when conducted, they could significantly improve the efficiency of PEIR management. Therefore, the objective of this study is to investigate the prevalence of PEIR and its related factors, and to evaluate its radiographic characteristics using CBCT.

## Methods and material

CBCT images of 590 unerupted teeth from 380 patients have been obtained from the 3D Accuitomo® 170 (Morita Corp., Japan) and retrieved from the archives (January 2018 – June 2021) of the Faculty of Dentistry, Mahidol University, Bangkok, Thailand. The study was performed according to the principles of Helsinki’s declaration. The protocol was reviewed and approved by the Ethical Review Board of Mahidol University (COA. No. MU-DT/PY-IRB 2021/076.2508).

### CBCT selection

CBCT images obtained from individuals who possessed at least one unerupted tooth encompassing supernumerary, impacted, or embedded teeth, which exhibited complete bony coverage, were recruited into the study. All unerupted teeth must undergo development beyond the cementoenamel junction (CEJ) level, signifying complete crown formation and mineralization. CBCT images from individuals with a history of systemic diseases, developmental dental anomalies, unerupted teeth associated with cysts, and benign or malignant tumors were not included. Furthermore, images characterized by inadequate quality, blurring, or other severe artifacts were excluded from the analysis.

### Data collection

Patient information, comprising age, sex and the specific data related to unerupted teeth, was systematically recorded. Additionally, details regarding the treatment planning for unerupted teeth, such as surgical removal, artificial eruption, and tooth transplantation, were documented for subsequent analysis.

All CBCT images were analyzed using the i-Dixel imaging software (Morita Corp., Japan). In each CBCT image, the field of view used was from 60 mm x 60 mm to 170 mm x 120 mm, and the voxel size ranged from 0.125 mm to 0.250 mm. The observers were able to enhance the image freely by adjusting the radiographic contrast, brightness, and magnification. The images were assessed in the multiplanar and cross-sectional view at the slice thickness of 1 mm. The images were displayed on the same computer monitor with a 27-inch screen size and 2560 × 1440 pixel resolution (Dell Inc., USA).

All images underwent assessment by three observers (P.A., R.A., and K.S.) for unerupted teeth associated with PEIR lesions, using six parameters: PEIR score, PEIR location, the number of lesions within the affected tooth, tooth angulation, tooth position, and pericoronal space. The presence of PEIR lesions was documented and compared among all three observers to ascertain the prevalence of lesions. A calibration process involved 20% of all subjects, randomly selected and evaluated by two oral and maxillofacial radiologists with over ten years of experience. Intra-rater reliability was assessed over a two-week period, and any discrepancies were resolved through consensus among all observers involved in this research.

### PEIR score

**Fig. 1:**
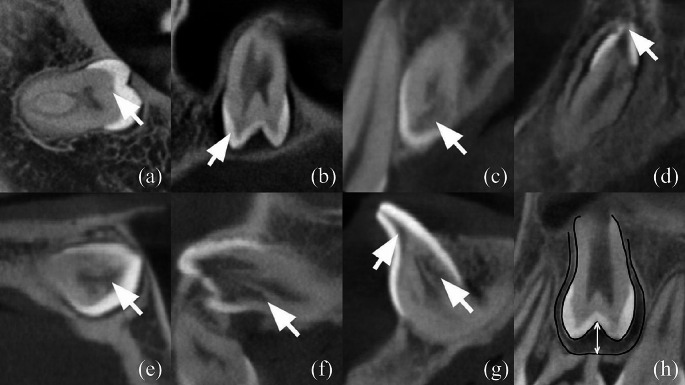
PEIR classification from CBCT images. (**a**) PEIR Score I, (**b**) PEIR Score II, (**c**) PEIR Score III, (**d**) PEIR Score IV, (**e**) PEIR Score V, (**f**) Root Extension, (**g**) 2 Lesions in 1 Affected Teeth, (**h**) Distance for Pericoronal Space Measurement

A modified classification of PEIR lesions was employed by combining the classifications proposed by Ozden and Acikgoz et al. and Demirtas et al. [11, 12]. The PEIR score was assigned based on the assessment of the degree of involvement of the crown in a three-dimensional view, as depicted in Fig. 1. The novel classification was introduced depending on the severity of the PEIR lesion as follows: score I indicates the extent of the lesion is less than one-third of the dentinal layer (Fig. 1a), score II denotes the extent of the lesion is between one-third and two-thirds of the dentinal layer (Fig. 1b), score III showing the extent of the lesion is more than two-thirds of the dentinal layer (Fig. 1c), score IV representing the extent of the lesion involves enamel (Fig. 1d), and score V signifying the extent of the lesion involves the pulpal tissue (Fig. 1e). PEIR lesions with higher PEIR scores reflect a higher severity of the lesion.

### PEIR location and number of lesions within the affected tooth

The PEIR location and the number of lesions within the affected tooth were examined along three axes. Consequently, the PEIR location was documented across seven positions, including buccal, lingual, mesial, distal, occlusal, cervical, and central. Moreover, cervical PEIR lesions which extended below the cementoenamel junction (CEJ) were recorded as the presence of root extensions.

### Tooth angulation and position

The tooth angulation and position of unerupted teeth were evaluated. Tooth angulation was determined by the angulation of the tooth itself according to Winter’s classification [7] and was subsequently recorded as mesioangular, distoangular, vertical, horizontal, buccoversion, linguoversion, or inverted. The tooth position was dependent on the tooth’s location in relation to its surrounding reference structure (edentulous space or adjacent tooth) and was then recorded as central, buccal or lingual, mesial or distal.

### Pericoronal space

For each unerupted tooth, the measurement of pericoronal space followed the methodology outlined by Wang et al. [13]. Briefly, the widest distance from the crown periphery of the follicle was measured, as illustrated in Fig. 1. A novel classification system for pericoronal space was proposed by modifying Wang’s Classification into five classes: class 0 denoting the absence of pericoronal space, class I indicating a pericoronal space of less than 1 mm, class II ranging from 1.00 mm to less than 2.00 mm, class III spanning from 2.00 mm to less than 3.00 mm, and class IV representing 3.00 mm or more. It is imperative to emphasize that a pericoronal space exceeding 3.00 mm (beyond class IV) suggested a pathological condition such as odontogenic cysts or tumors [9], warranting the exclusion of class IV from the study. Consequently, the analysis was confined to data falling within classes 0, I, II, or III.

### Data analysis

Statistical analysis was conducted using IBM SPSS Statistics for Windows, Version 27.0 (IBM Corp., Armonk, NY). Descriptive analysis was performed to ascertain the prevalence and associated factors of PEIR, encompassing patient age and sex, tooth type of the unerupted tooth, and PEIR location. Binary logistic analysis was employed to assess the presence of PEIR and its severity (PIER score) in conjunction with the patient’s demographic data and related factors. A *p*-value less than 0.05 was considered as statistical significance. Inter-rater and intra-rater reliability were evaluated through Cohen’s kappa test, yielding the following kappa values: presence of PEIR (1.00), number of lesions (1.00), PEIR score (0.78), location of lesion (0.85), Winter’s angulation (0.80), ectopic location (0.89), and pericoronal space (0.88), indicating that the interrater reliability ranges from substantial to almost perfect.

## Results

In total, 380 patients with 590 unerupted teeth were included in this study. It consisted of 139 males and 241 females at the age of 7 to 69 years. Of these, 76 patients (32 Males and 44 Females) were found to have PEIR, giving the subject prevalence of 20.0%. In addition, 80 teeth exhibited PEIR, reflecting tooth prevalence of 13.6%. The prevalence of PEIR was higher in men, at 23.0%, than females, at 18.3%. Notably, the subject prevalence in patients older than 20 years old was also higher than in those aged 20 or younger, at 24.0% and 14.9%, respectively.

**Fig. 2:**
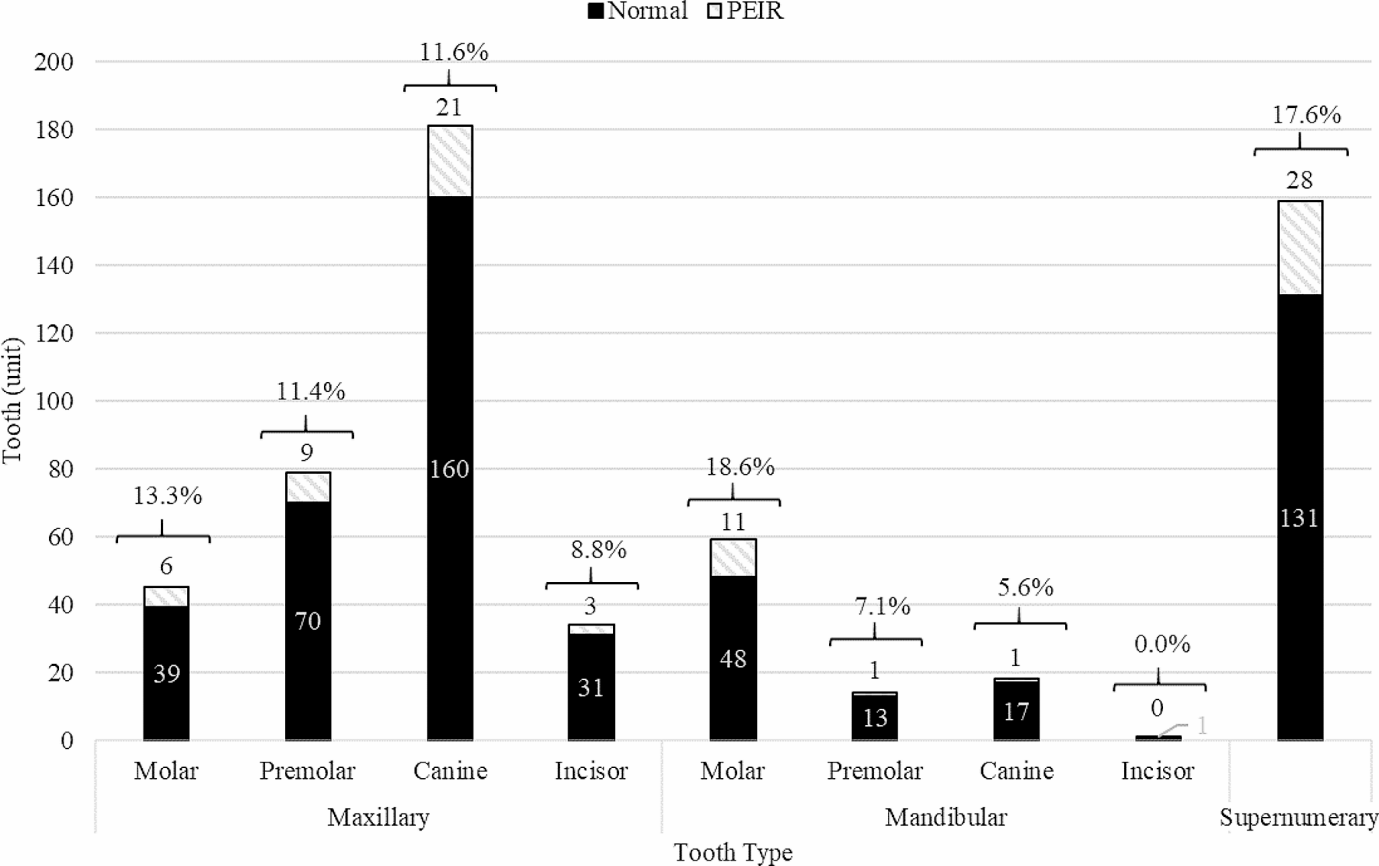
Summary of tooth prevalence within each tooth type

The tooth prevalence within each tooth type is illustrated in Fig. 2. Mandibular molars had the highest PIER prevalence at 18.6%, followed by supernumerary teeth at 17.6%, maxillary molars at 13.3% and maxillary canine at 11.6%. Moreover, PEIR was identified in 19.2% of unerupted teeth planned for artificial eruption or tooth transplantation (canines and molars). 59% of all lesions were categorized as PEIR score I (less than one-third of dentine thickness from the dentino-enamel junction), as shown in Table 1. Concerning to PEIR location, 76.2% of the lesions were found at the occlusal third of the crown, 19.3% at the lingual, and 9.6% at the buccal location.

**Table 1:** Summary of PEIR score and its prevalence

Tooth type		PEIR score (%) (*n*/*N*)				
		I	II	III	IV	V
Mx	Molar	83.3% (5/6)	0.0% (0/6)	0.0% (0/6)	16.7% (1/6)	0.0% (0/6)
	Premolar	70% (7/10)	10% (1/10)	0.0% (0/10)	20.0% (2/10)	0.0% (0/10)
	Canine	77.3% (17/22)	0.0% (0/22)	0.0% (0/22)	13.6% (3/22)	9.1% (2/22)
	Incisor	25% (1/4)	0.0% (0/4)	0.0% (0/4)	0.0% (0/4)	75.0% (3/4)
Md	Molar	100% (11/11)	0.0% (0/11)	0.0% (0/11)	0.0% (0/11)	0.0% (0/11)
	Premolar	100% (1/1)	0.0% (0/1)	0.0% (0/1)	0.0% (0/1)	0.0% (0/1)
	Canine	0.0% (0/1)	0.0% (0/1)	0.0% (0/1)	100% (1/1)	0.0% (0/1)
	Incisor	N/A				
Supernumerary		25% (7/28)	3.6% (1/28)	7.1% (2/28)	25% (7/28)	39.3% (11/28)
Total		59.0% (49/83)	2.4% (2/83)	2.4% (2/83)	16.9% (14/83)	19.3% (16/83)

^n = teeth with specific PEIR characteristics, N = total teeth with presence of PEIR, Mx = Maxillary, Md = Mandibular, N/A = not available^

^For PEIR score; I = less than 1/3 of dentin level, II = between 1/3 and 2/3 of dentin level, III = more than 2/3 of dentin level, IV = enamel involvement, V = pulpal involvement^

Binary logistic analysis was employed to assess the presence of PEIR in relation to the patient’s demographic data and related factors. The presence of PEIR was associated with tooth angulation and position as detailed in Table 2. Transverse and inverted angulation presented a higher likelihood of developing PEIR than vertical angulation. Moreover, central tooth position exhibited a higher likelihood of having PEIR when compared to buccal tooth position.

**Table 2:** Results of binary logistic regression analysis between patient’s demographic data and related factors and presence of PEIR

Demographic Data and Related Factors	β	S.E.	Odds Ratio	95% CI of Odds Ratio	*p*-value
**Sex**					
Male	-	-	1	Reference	
Female	0.059	0.405	1.060	[0.479–2.347]	0.885
**Age Group**					
< 20	-	-	1	Reference	
≥ 20	0.150	0.405	1.162	[0.526–2.569]	0.710
**Tooth type**					
Anterior	-	-	1	Reference	
Posterior	0.451	0.425	1.570	[0.682–3.613]	0.289
Supernumerary	2.038	0.671	7.677	[2.061–28.602]	0.002
**Tooth Angulation**					
Vertical	-	-	1	Reference	
Mesioangular	0.347	0.522	1.414	[0.509–3.931]	0.506
Horizontal	0.517	0.560	1.677	[0.560–5.023]	0.355
Distoangular	0.144	1.030	1.154	[0.153–8.691]	0.889
Buccoversion	-0.196	1.269	0.822	[0.068–9.878]	0.877
Linguoversion	-0.079	0.899	0.924	[0.159–5.378]	0.930
Transverse	1.936	0.835	6.934	[1.351–35.601]	0.020
Inverted	1.729	0.821	5.636	[1.128–28.174]	0.035
**Tooth Position**					
Buccal	-	-	1	Reference	
Central	1.198	0.591	3.313	[1.040–10.549]	0.043
Mesial	-0.620	1.214	0.538	[0.050–5.814]	0.610
Distal	0.794	1.073	2.211	[0.270–18.099]	0.459
Lingual	0.910	0.631	2.485	[0.722–8.555]	0.149
**Pericoronal Space**					
Class 0	-	-	1	Reference	
Class I	-0.312	0.597	0.732	[0.227–2.359]	0.601
Class II	-0.945	0.629	0.389	[0.113–1.332]	0.133
Class III	-0.973	0.720	0.378	[0.092–1.550]	0.177
Class IV	-2.298	1.312	0.100	[0.008–1.314]	0.080

The most severe form of PEIR, indicated by PEIR score IV and V, were evaluated using binary logistic analysis in association with the patient’s demographic data and related factors, as shown in Table 3. For tooth angulation, lingual, distal and inverted angulation were found to have a higher likelihood of severe PEIR compared to vertical angulation. Additionally, according to pericoronal space classification, the presence of pericoronal space of class II significantly reduced the likelihood of severe PEIR compared to pericoronal space of class 0 (absence of pericoronal space).

**Table 3:** Results of Binary Logistic Regression Analysis between Patient’s Demographic Data and Related Factors and PEIR Score IV and V

Demographic Data and Related Factors	β	S.E.	Odds Ratio	95% CI of Odds Ratio	*p*-value
**Sex**					
Male	-	-	1	Reference	
Female	-0.625	0.850	0.535	[0.101–2.833]	0.462
**Age Group**					
< 20	-	-	1	Reference	
≥ 20	0.787	0.930	2.196	[0.355–13.586]	0.398
**Tooth type**					
Anterior	-	-	1	Reference	
Posterior	-3.558	1.915	0.028	[0.001–1.215]	0.063
Supernumerary	0.656	1.068	1.927	[0.238–15.615]	0.539
**Tooth Angulation**					
Vertical	-	-	1	Reference	
Mesioangular	1.099	1.749	3.001	[0.097–92.527]	0.530
Horizontal	1.946	1.588	7.003	[0.312–157.283]	0.220
Distoangular	7.327	3.122	1521.234	[3.349–691049.972]	0.019
Buccoversion	-18.757	40192.970	0.000	[0.000]	1.000
Linguoversion	5.555	2.438	258.548	[2.176–30715.430]	0.023
Transverse	2.249	1.617	9.477	[0.399–225.349]	0.164
Inverted	2.756	1.343	15.730	[1.131–218.787]	0.040
**Tooth Position**					
Buccal	-	-	1	Reference	
Central	-2.132	2.131	0.119	[0.002–7.723]	0.317
Mesial	-22.804	40192.970	0.000	[0.000]	1.000
Distal	2.637	3.342	13.977	[0.020–9774.714]	0.430
Lingual	-2.893	2.113	0.055	[0.001–3.487]	0.171
**Pericoronal Space**					
Class 0	-	-	1	Reference	
Class I	0.015	0.849	1.015	[0.192–5.358]	0.986
Class II	-3.503	1.673	0.030	[0.001–0.799]	0.036
Class III	-19.101	11401.844	0.000	[0.000]	0.999
Class IV	-17.488	40192.970	0.000	[0.000]	1.000

## Discussion

Throughout this investigation, demographic data of patients and various associated factors were examined, revealing a discernible correlation with PEIR. The manifestation of this lesion was particularly conspicuous among patients aged 20 years and above, where unerupted teeth were observed to be impacted or embedded [11, 14]. These findings aligned notably with the proposed etiological framework of PEIR, specifically the resorptive theory. As posited by Seow & Hackley, the heightened pressure experienced by unerupted teeth may compromise the integrity of the reduced enamel epithelium, thereby activating resorptive cells to engage in dentin resorption [4]. Furthermore, distinct characteristics of unerupted teeth, such as tooth angulation and position, demonstrate a noticeable association with the presence of PEIR. The existence of obstructive forces in the eruption pathway, contributing to tooth impaction, may precipitate the progression of these cases into PEIR [3].

The prevalence of PEIR among Thais in this study (20.0%) obviously contrasts with the findings of the study conducted by Manmontri et al. (1.63%). This divergence within the same population could be elucidated by variations in research methodologies. Manmontri et al. utilized panoramic radiographs, while the present study employed CBCT images. The present findings indicated that PEIR lesions were not confined solely to the mesio-distal dimension but also extend to the bucco-lingual dimension. While panoramic radiographs offer superior visualization of lesions in the mesio-distal view, they exhibit limitations in localizing lesions in the bucco-lingual view. In contrast, CBCT images proficiently demonstrate lesions in all dimensions, affirming the precision of lesion localization with CBCT over panoramic radiographs. This clarification accounts for the pronounced discrepancy in PEIR prevalence observed in the present study compared to those documented by Manmontri et al. Furthermore, in comparison to another CBCT study, Demirtas et al. reported a subject prevalence of PEIR of 15.1%, which aligned with the present findings. This underscored the pivotal role of CBCT as a diagnostic aid in localizing and assessing the severity of PEIR lesions. However, CBCT is not a modality that is routinely prescribed due to concerns about radiation exposure, in accordance with the As Low As Diagnostically Acceptable (ALADA) principle. This suggests that the images acquired were prescribed for patients who require additional investigation of impacted or embedded teeth.

One of the factors influencing PEIRs detection is the selection of voxel size. Regions smaller than the voxel size may remain undetected in CBCT due to the partial volume averaging effect [15]. Our study included the field of view from 60 mm x 60 mm to 170 mm x 120 mm, and the voxel size ranged from 0.125 mm to 0.250 mm. PEIRs were detected in all voxel sizes, including the largest voxel size of 0.250 mm. This suggests that the sizes of the PEIR lesions were large enough to be seen in the large field of view. Therefore, the detection of PEIRs should be considered in all cases during general CBCT interpretation.

The clinical significance of this study was underscored by the verified association between PEIR characteristics and patient demographic data, as well as related factors. This finding proved valuable in the diagnosis and evaluation of PEIR within clinical settings. Regarding tooth position and angulation, central tooth positioning, along with transverse or inverted angulation, are identified as influential factors contributing to the occurrence of PEIR. Therefore, unerupted teeth displaying these characteristics should undergo careful screening for the presence of PEIR. The clinical relevance of this association is evident in the management of unerupted teeth. Numerous considerations must be weighed before opting for removal or non-removal interventions. While the presence of pathology may suggest extraction, this is not always the case for PEIR. Treatment options for PEIR range from restorative interventions before or after eruptions to tooth extraction in extensive cases [4, 12]. Extraction is only indicated if the lesion is symptomatic or has progressed extensively. Therefore, unerupted teeth with mild to moderate PEIR lesions could be retained through artificial tooth eruption or might serve as candidates for tooth transplantation. Early diagnosis of PEIR is crucial and clinically significant for treatment, enabling immediate intervention and enhancing prognosis.

The results of this study revealed the highest prevalence of PEIR in mandibular molars. It is recommended that screening for PEIR in posterior teeth should be conducted in the occlusal third, where more than 83.3% of premolars and molars with PEIR were found to have a lesion located in this position. Currently, there is no knowledge available to explain this association, making it an intriguing topic for further studies.

Previous study indicated that an unerupted tooth, regardless of PEIR, typically maintained an average pericoronal space of less than 2 mm [14], in accordance with the findings of the present study. Significantly, a class 0 pericoronal space (absence of pericoronal space) showed a 3.5 times higher likelihood of developing a severe form of PEIR compared to in class II (1 mm to less than 2 mm). The association between the pericoronal space and PEIR also supported the resorptive theory [4]. A reduction in the pericoronal space leads to an increase in internal pressure, subsequently inhibiting the formation of dentin and, in turn, triggering the resorption of dentin. Hence, a more thorough examination for PEIR is recommended.

While conventional radiographs are not ideal for the screening of PEIR due to the limitations of two-dimensional images, it is recommended to incorporate regular PEIR screening when panoramic radiographs are routinely prescribed [9]. The ALADA principle should be considered along with the diagnostic benefits of CBCT. It should be utilized only when PEIR is suspected or when additional information is required. Suspected teeth are those exhibiting the characteristics mentioned earlier, and advanced radiographic imaging can be employed to precisely analyze and interpret these lesions, thereby preventing misdiagnosis and facilitating the development of the most effective treatment plan with the most favorable outcome. We recommend selecting the smallest field of view available on the CBCT unit, as it results in a smaller effective dose [16]. Moreover, a recent study by Oenning et al. [17] recommended the dose reduction technique combined with small voxel size selection, which can reduce the effective dose by half while maintaining good image quality.

The limitation of this study is that the progression of PEIR lesions was not evaluated, given that it was a cross-sectional study. Nature of cross-sectional studies do not investigate the static or progressive nature of the lesion. A long-term follow-up may be necessary to assess the progression rate of PEIR lesions. Previous authors, recognizing the urgency in identifying and managing progressive PEIR, have emphasized the need for such evaluations [11]. Depending on the diagnostic outcome, treatment protocols for PEIR have been established. Clinical management of the lesions is intricate, involving considerations such as root development, the necessity for pulp therapy, the anticipated lifespan of the non-vital tooth, and the decision to retain the tooth [9].

Although factors like the size of the lesion were examined in this study, the rate of progression and the expected time of eruption could not be determined due to the limitations of study design. While a previous study utilizing panoramic radiographs indicated that 89.1% of PEIR lesions were static over an average follow-up period of 36 months [18], it may be prudent to assume that more than the remaining 10% display progressive characteristics, warranting urgent intervention, given the constraints posed by two-dimensional images.

This study has identified factors influencing the screening of unerupted teeth for PEIR and proposes the following clinical recommendations:

1. Observation for PEIR in Kept Unerupted Teeth
  When the decision is made to retain an unerupted tooth, it is recommended to regularly observe it for the presence of PEIR.
2. Close inspection for specific characteristics
  Unerupted teeth displaying transverse angulation, inverted angulation, central tooth position, or an absence of pericoronal space should undergo thorough inspection for potential PEIR.
3. Awareness of tooth type prevalence
  While PEIR can be found in all tooth types, its occurrence is particularly common in maxillary and mandibular molars.
4. Regular Observation with radiographs
  It is recommended to consistently observe for PEIR when conventional radiographs are taken, as they are routinely prescribed. The use of CBCT is advised only when PEIR is suspected or additional information is deemed necessary.

## Conclusion

PEIR represents a resorptive abnormality affecting unerupted teeth. This study reports a 20% subject prevalence of PEIR. Among the teeth that were found PEIR lesion, there is a prevalence of 19.2% in unerupted teeth that were planned for artificial eruption or tooth transplantation, specifically in canines and molars. The utilization of CBCT can serve as a valuable tool, aiding clinicians in the comprehensive evaluation and formulation of a treatment plan for unerupted teeth exhibiting PEIR.

Particular attention should be given to unerupted teeth demonstrating transverse or inverted-angulated orientation, a central tooth position, and the absence of pericoronal space, especially in molars, as these characteristics heighten the suspicion of PEIR. Early detection of PEIR is of paramount importance, facilitating the development of a thorough and suitable treatment plan, with a focus on the preservation of these affected teeth for optimal patient outcomes.

## Data Availability

No datasets were generated or analysed during the current study.
